# A consultation-level intervention to improve care of frequently attending patients: a cluster randomised controlled feasibility trial

**DOI:** 10.3399/bjgpopen18X101623

**Published:** 2019-01-09

**Authors:** Rebecca K Barnes, Helen Cramer, Clare Thomas, Emily Sanderson, Sandra Hollinghurst, Chris Metcalfe, Sue Jackson, Charlie Record, Helen Thorley, David Kessler

**Affiliations:** 1 Senior Research Fellow, Population Health Sciences, Bristol Medical School, University of Bristol, Bristol, UK; 2 Research Fellow, Population Health Sciences, Bristol Medical School, University of Bristol, Bristol, UK; 3 Senior Research Associate, Population Health Sciences, Bristol Medical School, University of Bristol, Bristol, UK; 4 Research Associate, Bristol Randomised Trials Collaboration (BRTC), Population Health Sciences, Bristol Medical School, University of Bristol, Bristol, UK; 5 Senior Lecturer in Health Economics, Population Health Sciences, Bristol Medical School, University of Bristol, Bristol, UK; 6 Professor of Medical Statistics, Population Health Sciences, Bristol Medical School, University of Bristol, Bristol, UK; 7 Professor of Medical Statistics, Bristol Randomised Trials Collaboration (BRTC), Population Health Sciences, Bristol Medical School, University of Bristol, Bristol, UK; 8 Visiting Lecturer, Health & Social Sciences, University of the West of England, Bristol, UK; 9 Partner, Frome Valley Medical Centre, Bristol, UK; 10 Research Associate, Efficiency Team, The National Institute for Health Research Collaboration for Leadership in Applied Health Research and Care West (NIHR CLAHRC West), Bristol, UK; 11 Reader in Primary Care, Population Health Sciences, Bristol Medical School, University of Bristol, Bristol, UK

**Keywords:** Frequently Attending Patients, General Practice, Continuity of Patient Care, BATHE technique, Health Communication, Feasibility Studies

## Abstract

**Background:**

Frequent attenders (FAs) to primary care receive considerable NHS resources without necessarily gaining benefit, and may even be harmed.

**Aim:**

To assess the feasibility of a consultation-level intervention to improve care and address service use of FAs.

**Design & setting:**

A cluster randomised controlled feasibility trial was undertaken. The study used a mixed-methods process evaluation and took place in six practices in England.

**Method:**

All practices screened the top 3% of all attending patients over the previous 12 months for eligibility. Following randomisation, intervention patients were matched with named GPs, trained to use the Background, Affect, Trouble, Handling, Empathy (BATHE) technique during consultations. Telephone consultations were encouraged. Feasibility outcomes assessed were recruitment, retention, data collection and completeness, implementation fidelity, and acceptability

**Results:**

A total of 599/1328 (45.1%) FAs were eligible. Four practices were randomised to the intervention (*n* = 451) and two to usual care (*n* = 148). A total of 96 (23.7%) patients were recruited to complete questionnaires. Retention and completeness of data were good; for example, 76% of those agreeing to complete questionnaires did so at the 12-month assessment point. Thirty-four GPs were trained and delivered BATHE ≥1 times to 50.1% of patients (*n* = 577 consultations). There were minimal increases in continuity and telephone consultations. Patients were positive about the intervention, but noticed little change in their care. Despite valuing BATHE, low adherence to training was indicated and GPs used it less than anticipated.

**Conclusion:**

It was feasible to identify FAs and collect trial data. GPs were keen to engage and there was evidence that the BATHE technique was taken into practice. Optimising training is likely to improve fidelity. The intervention was low cost and low risk.

## How this fits in

FAs in primary care are a heterogenous group with multiple reasons for attending: physical, psychological, and social. Doctors report caring for FAs to be challenging and high service use risks iatrogenic harm to patients. This consultation-level intervention moves away from patient ‘treatment’ towards what patients and clinicians can do together to improve recognition and support for healthcare problems and related needs. The aim is to increase patient independence over time.

## Introduction

Amid concerns about the sustainability of UK primary care services,^1^ the issue of individuals attending more often than might be expected is becoming more visible. The top 3% of attenders to primary care have been associated with 15% of overall appointments.^2^ Moreover, FAs have consistently been shown to have higher investigation, referral, and hospital admission rates than non-FAs.^3^

Different definitions of FAs are used throughout the literature, which are based on frequency, duration, or both.^4^ However, FAs comprise a heterogeneous group with a wide variety of needs.^2,5,6^ Just over 50% are reported as having physical disease, and, in more than 50% of cases, social factors (such as low social support, unemployment, and divorce) are apparent. Multiple problems (physical, psychological, and social) are reported in one-third of cases.^4^

Prior research has shown that frequent attendance can be driven by disease or patient factors,^2^ or professional and organisational factors.^7^ However, interventions are often patient-focused.^8^ Measures of change have been varied, including mental health symptom severity, quality-of-life indicators, morbidity, or consultation frequency. Systematic reviews consistently report uncertainty over the effectiveness of interventions, concluding that this may in part be owing to the heterogenous nature of FAs.^9,10^ One suggestion has been to move away from patient treatments and towards consultation-level changes.^10^

The research question was: ‘Is it feasible to run a randomised controlled trial of a consultation-level intervention for FAs in primary care?' The objectives were to:

1. evaluate recruitment and retention capability, and resulting sample characteristics;
2. evaluate and refine data collection procedures and outcome measures;
3. assess the extent to which the intervention could be implemented as planned; and
4. assess the acceptability of the intervention to staff and patients.

## Method

### Study design

A cluster randomised feasibility trial was undertaken, with an economic evaluation and mixed-methods process evaluation.

### Procedure

#### Practices

Six general practices with differing levels of neighbourhood deprivation were recruited via the Clinical Research Network: West of England. Practice eligibility criteria were having the structural and/or organisational capacity to participate, and a list size of ≥ 7000 patients.

#### Screening

All practices screened electronic medical records (EMRs) to identify eligible patients. The eligibility criteria were as follows: currently registered at a study practice; aged ≥18 years; and falling into the top 3% of attenders over the previous 12 months. All types of GP contacts were included. Study GPs excluded the following: patients whose level of consulting could be accounted for by diagnosed physical or mental illness; patients with life-threatening illness such as cancer; patients aged >80 years with ≥4 medical problems; patients at high risk of hospital admission; patients undergoing distressing life events; vulnerable adults; and patients without capacity to provide informed consent.

It was anticipated that ≥40 eligible patients would be identified per average size practice. To enable sufficient testing of procedures, it was planned that 15 patients would be recruited from six practices, which would give a total sample of 90 patients. Study data were collected and managed using REDCap hosted at the University of Bristol.

#### Randomisation

Practices were randomised by a member of the research team not involved in recruitment. Practices were divided into two blocks of three similar in terms of deprivation and list size (further information available from the authors on request). Within each block, two practices were randomly allocated to the intervention group and one to the control group to enable more exploratory testing of the intervention.

#### Patient recruitment

Eligible patients in both groups received an information sheet and consent form inviting them to enrol in a questionnaire study. Patients wishing to participate returned a signed consent form and a completed baseline questionnaire to the study team in a prepaid envelope. Those not wishing to take part were asked to return a blank questionnaire. Intervention practices also sent a letter (further information available from the authors on request) informing eligible patients that the practice would be making some changes to their care.

### Intervention and control conditions

The intervention was designed at a local practice with help from patients. The design was based on the assumption that increased continuity of care and enhanced GP consultation skills (to elicit the wider context to patients’ problems and support self-management) would reduce high consultation rates and NHS costs.

Intervention practices assign all eligible patients a named GP trained to use the BATHE technique (Box 1). Based around a series of linked questions, BATHE supports a person-focused approach to consulting. It elicits the wider context to patients’ problems, and can act as an informal screen for emotional distress, support self-management, and strengthen clinician–patient relationships.^11^

**Box 1:** The BATHE consultation technique^11^

	BATHE is an acronym pertaining to a series of four linked questions and a closing statement as given below: B = Background Question 1. What is going on in your life? A = Affect Question 2. How do you feel about that? T = Trouble Question 3. What about the situation troubles you the most? H = Handling Question 4. How are you handling that? E = Empathy Closing Statement. That must be difficult for you (or something of an appropriately similar nature).

A 30-minute training session was delivered to reception staff, with top-up training at 6 months. Staff were trained to ask for the patient’s name first during appointment-making and to offer the option of a telephone consultation. To facilitate matching, a ‘pop-up’ alert was placed on EMRs identifying study patients and their named GPs. A 1 hour-long training session was delivered to study GPs by an experienced BATHE trainer, with two further 1-hour top-up sessions, 4 and 7 months later. All training sessions were video-recorded.

BATHE training consisted of the following: an overview of the trial; a talk introducing the technique and its underlying principles; and an opportunity to role-play and to ask questions. GPs were given a prompt card to place in their consulting rooms and encouraged to practise the technique. GPs were asked to incorporate BATHE into all consultations with eligible patients (where appropriate) and to document its use in the EMR using a dedicated Read code.

Control practices were asked to continue with usual care.

### Outcomes

All eligible patients were followed-up for 12 months and data collected on consultation rate and length (EMR review). Data were collected from questionnaire study patients at baseline, 3, 6, and 12 months on the following: health-related quality of life (EQ-5D-5L);^12^ physical and mental health (Short Form health survey [SF-12]);^13^ anxiety symptom severity (Generalised Anxiety Disorder [GAD-7]);^14^ depression symptom severity (Patient Health Questionnaire [PHQ-9]);^15^ somatic symptom severity (Patient Health Questionnaire [PHQ-15]);^16^ self-management (Patient Activation Measure [PAM13]);^17^ satisfaction with care and trust (GP Patient Survey).^18^

A £5 shopping voucher was offered for each completed questionnaire returned. Follow-up calls were used to collect further details about health-related resource use and personal costs at 6 and 12 months. Permissions were also sought to extract data on prescriptions and tests from EMRs at 12 months.

### Process evaluation

The feasibility of identifying eligible patients was assessed using recorded semi-structured interviews with GPs in all practices following screening. Implementation fidelity was assessed using observations of appointment-making; 6-weekly practice data audits of the extent of matching and use of the BATHE code; and consultation recordings at 3 and 6 months. Acceptability was assessed by training observations and recorded semi-structured interviews with GPs, staff, and patients post-intervention.

### Data analysis

Study data were collected and managed using REDCap hosted at the University of Bristol. Recruitment and retention data were presented as a CONSORT flow chart. Summary statistics for each group were presented for the questionnaires at baseline, 3, 6, and 12-month assessment points; consultation rates; and fidelity measures. Intervention costs were estimated using data on training, screening, any extra consultations costs, and sundry consumables. Relevant unit costs for the intervention (practice-level), other NHS resource use, and wider societal costs were identified from established national UK sources.^19,20,21^ All costs were reported in pounds sterling at 2016 prices. The mean utilities and quality-adjusted life years accrued, by group, were estimated from responses to the EQ-5D-5L and reported for all complete cases. Stata (version 14) was used to perform all statistical analyses.

Field notes from observations and interview data transcripts were analysed using an agreed thematic framework,^22^ which was based on the feasibility study aims. Consultation recordings were transcribed and analysed according to conversation analytic methods,^23^ focusing on GP delivery of and patient responses to the BATHE intervention.

## Results

### Patient recruitment and retention

**Figure 1:**
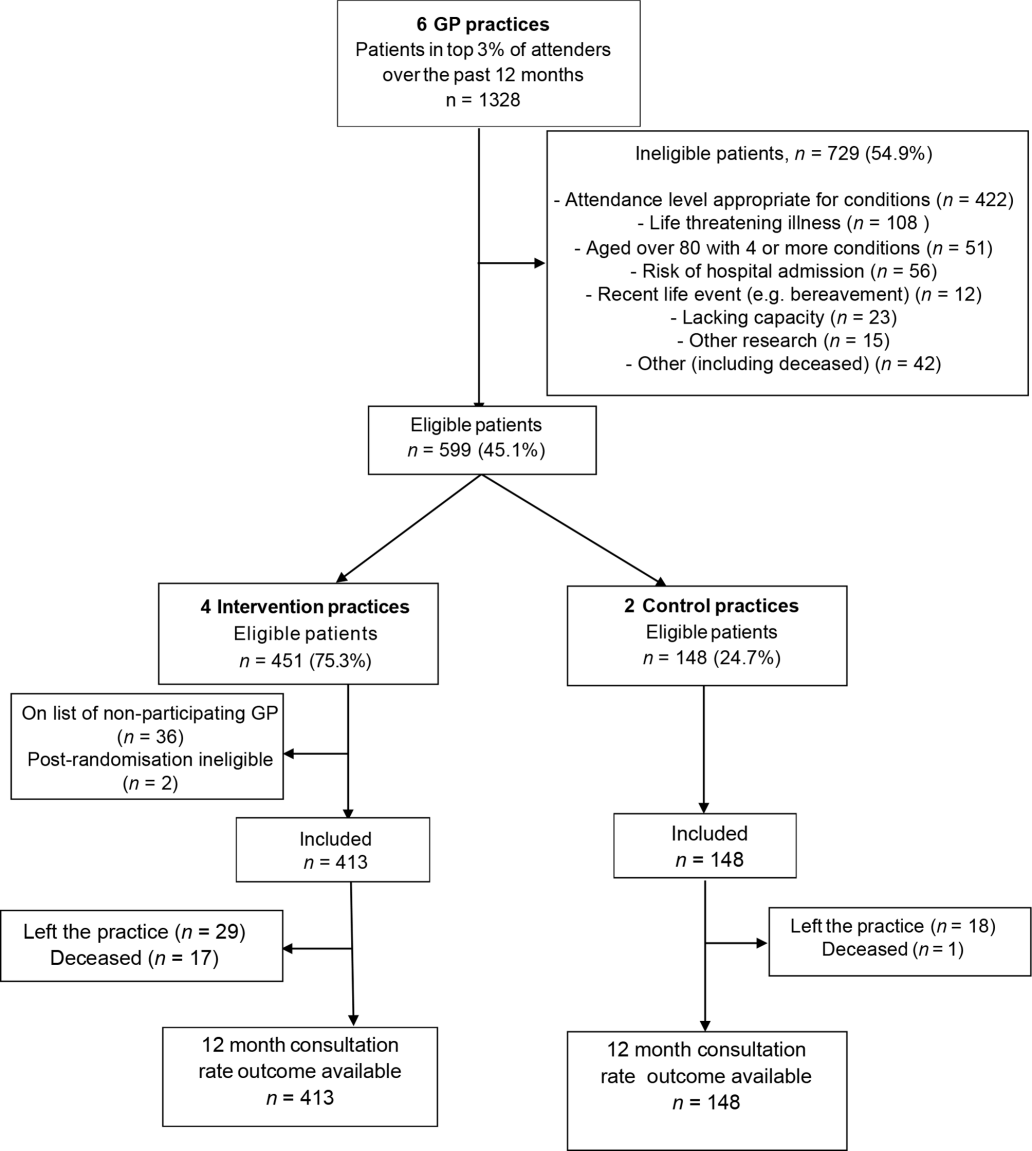
CONSORT flowchart: all eligible patients

**Figure 2:**
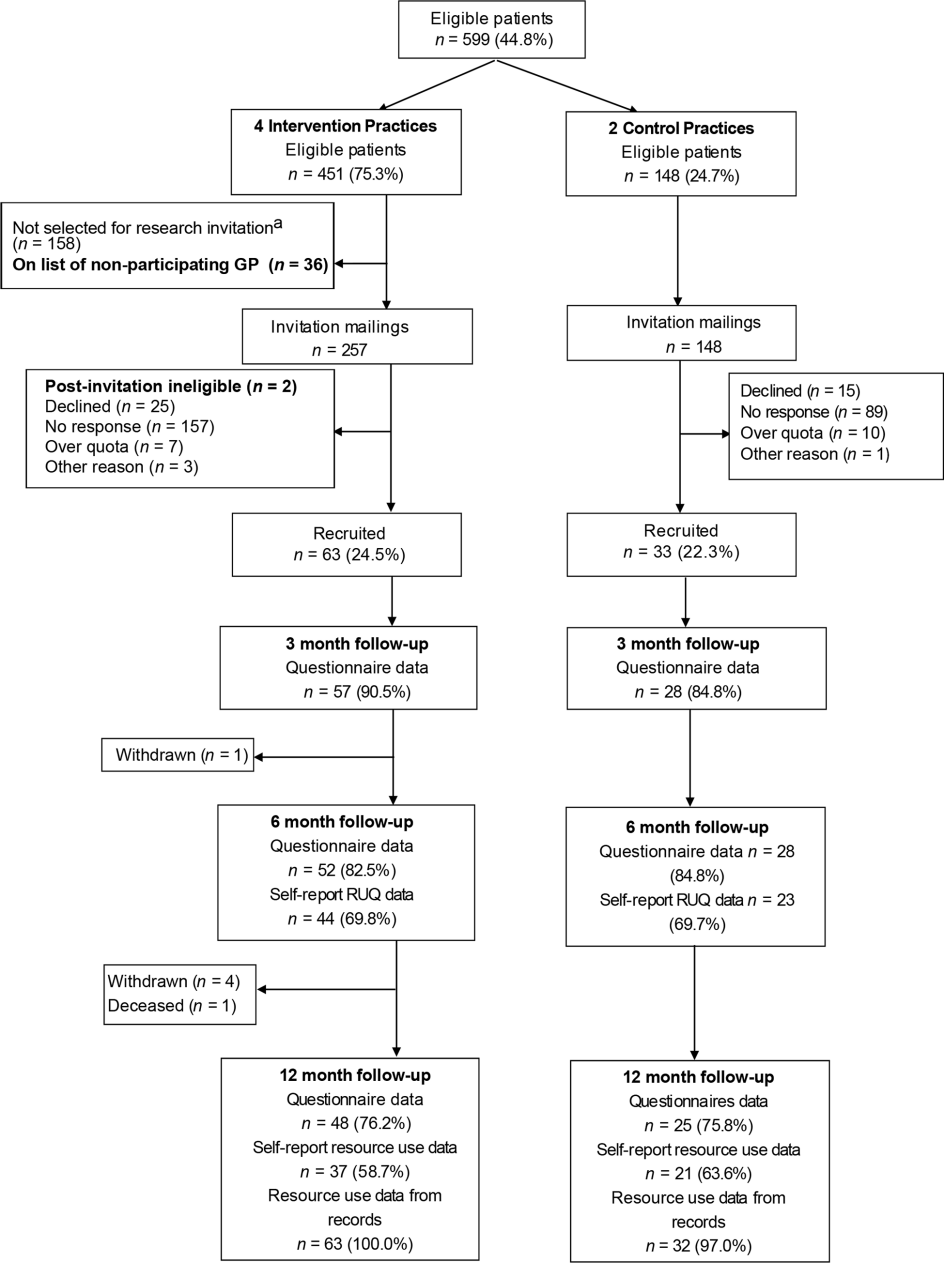
CONSORT flowchart: questionnaire study. ^a^In the larger practices, study invitations were only sent to half the eligible patients in order to avoid over-recruitment. RUQ = resource use questionnaire.

A total of 1328 patients made up the top 3% of attenders 12 months before the intervention across all practices. Following exclusions, 599 (45.1%) patients were deemed eligible. Four practices were randomised to the intervention group (451 patients) and two to usual care (148 patients), as shown in Figure 1. Eligible patients were recruited from July–October 2015. A total of 113/405 (27.9%) patients responded positively but, for practical reasons, recruitment was restricted to 96/405 (23.7%) patients (63 intervention, 33 control). Questionnaires were returned by 86 (89.6%) patients at 3 months, 80 (83.3%) at 6 months and 73 (76.0%) at 12 months. Where reported, additional service resource use data were collected by telephone from 67/78 (85.9%) patients at 6 months, and 58/69 (84.1%) at 12 months (Figure 2). Consultation rate data were collected at 12 months for 561 (93.7%) patients (*n* = 413 intervention, *n* = 148 control; Figure 1).

### Patient characteristics

Mean patient age in the intervention group was 58.7 years (SD 21.1, range 19–98), and 33.4% were male. Mean age in the control group was 47.5 years (SD 17.3, range 18–84), and 20.9% were male. For excluded patients the mean age was 60 years (SD 21.3, range 18–100), and 37.1% were male (further information available from the authors on request). Table 1 shows further demographic and clinical data from the questionnaire study patients.

**Table 1:** Questionnaire sample demographics

		Intervention, % (*n*)	Control, % (*n*)
Total patients		63	33
Mean age, years (SD) [range]		62.39 (20.57) [25–95]	57.75 (17.54) [20– 84]
Proportion male		28.57 (18)	36.36 (12)
Ethnicity proportion	White/ White British	92.06 (58)	96.97 (32)
	Other	6.35 (4)	0.00 (0)
	Not recorded	1.59 (1)	3.03 (1)
Living situation	Alone	33.33 (21)	24.24 (8)
	With someone	63.49 (40)	60.61 (20)
	Not reported	3.17 (2)	15.15 (5)
Patients in each quintile of IMD (lower quintile = more deprived)	Quintile 1	15.87 (10)	21.21 (7)
	Quintile 2	15.87 (10)	30.30 (10)
	Quintile 3	22.22 (14)	33.33 (11)
	Quintile 4	19.05 (12)	15.15 (5)
	Quintile 5	25.39 (16)	0.00 (0)
	Not recorded	1.59 (1)	0.00 (0)
Patients with different numbers of QOF conditions	Number of QOF conditions		
	0	42.86 (27)	36.36 (12)
	1	28.57 (18)	24.24 (8)
	2	17.46 (11)	21.21 (7)
	3	9.52 (6)	9.09 (3)
	4	1.59 (1)	3.03 (1)
	5	0.00 (0)	3.03 (1)
	Missing	0.00 (0)	3.03 (1)

IMD = Index of Multiple Deprivation. QOF = Quality Outcomes Framework.

The average number of QOF conditions per study patient across both groups was 1.07. Most common conditions: depression/schizophrenia (*n* = 20), anxiety/panic (*n* = 13), high blood pressure (*n* = 13), asthma (*n* = 12), COPD (*n* = 12).

### Questionnaire responses and consultation rates

In both groups the mean scores for all health-related measures improved very slightly over the 12 months (further information available from the authors on request). One of the functions of BATHE is to support patient self-management. Notably in both groups at baseline, the majority of PAM-13 (patient activation) scores fell in the lowest category: ‘People tend to be overwhelmed and unprepared to play an active role, they are predisposed to be passive recipients of care.’^24^ However, 31.9% intervention versus 20.8% control patients, had moved up one or more categories at 12 months (further information available from the authors on request).

Across all practices at 12 months, there was a drop in mean consultation rate from 22.8 to 14.9, a difference of 7.9 consultations (SD 11.62), suggesting some expected regression to the mean. Patients in the intervention arm had a 5.6% lower consultation rate (95% CI = -18.6 to 9.4) than patients in the control arm (Table 2).

**Table 2:** Comparison of consultation rates at 12-months post-randomisation

	Total consultations, *n*	Patients, *n*	Total patient years of follow-up	Mean consultation rate	95% CI	Rate ratio (95% CI based on a negative binomial regression)
Practice 2	2160	153	145.72	15.51	(13.77 to 17.47)	
Practice 3	528	33	30.97	17.37	(13.67 to 22.06)	
Practice 4	2239	164	153.76	15.56	(13.67 to 17.72)	
Practice 5	1024	63	61.34	18.53	(15.64 to 21.95)	
Practice 1	661	38	36.42	18.26	(14.30 to 23.32)	
Practice 6	1735	110	103.97	16.70	(14.33 to 19.47)	
Intervention	5951	413	391.78	16.14	(14.97 to 17.40)	0.944 (0.814 to 1.094)
Control	2396	148	140.39	17.10	(15.01 to 19.48)	

CI = confidence intervals.

### Process evaluation

A total of 34 GPs (further information available from the authors on request) attended ≥1 BATHE training sessions (the majority of GP partners and salaried GPs). At 12 months, in terms of reach, GPs recorded using BATHE in 577/5951 (9.7%) consultations (range 7.2–19.2). A total of 451/577 (78.2%) were face-to-face and 119/577 (20.6%) telephone. In terms of dose, the number of eligible patients exposed to BATHE ≥1 times was 207/413 (50.1%), as shown in Table 3a (range 36.4– 84.1; further information available from the authors on request).

**Table 3a:** Adherence to the intervention components

		Total, *n* (*n* = 413)	Practice 2, *n* (*n* = 153)	Practice 3, *n* (*n* = 33)	Practice 4, *n* (*n* = 164)	Practice 5, *n* (*n* = 63)
Eligible patients consulting with named GP, *n* (%)	0 times	102 (24.7)	17 (11.11)	3 (9.09)	73 (44.51)	9 (14.29)
	≥1 times	311 (75.3)	136 (88.89)	30 (90.91)	91 (55.49)	54 (85.71)
Eligible patients consulting by telephone, *n* (%)	0 times	82 (19.85)	39 (25.49)	3 (9.09)	36 (21.95)	4 (6.35)
	≥1 times	331 (80.15)	114 (74.51)	30 (90.91)	128 (78.05)	59 (93.65)
Eligible patients exposed to BATHE, *n* (%)	0 times	206 (49.88)	79 (51.63)	21 (63.64)	96 (58.54)	10 (15.87)
	≥1 times	207 (50.12)	74 (48.37)	12 (36.36)	68 (41.46)	53 (84.13)
Frequency of eligible patients exposed to BATHE, *n* (%)	Proportion of consultations					
	0%	206 (49.88)	79 (51.63)	21 (63.64)	96 (58.54)	10 (15.87)
	0.1–10.0%	54 (13.08)	22 (14.38)	3 (9.09)	26 (15.85)	3 (4.76)
	10.1–20.0%	70 (16.95)	23 (15.03)	5 (15.15)	21 (12.8)	21 (33.33)
	20.1–30.0%	40 (9.69)	15 (9.8)	2 (6.06)	6 (3.66)	17 (26.98)
	30.1–40.0%	21 (5.08)	8 (5.23)	1 (3.03)	5 (3.05)	7 (11.11)
	≥40.1%	22 (5.33)	6 (3.92)	1 (3.03)	10 (6.1)	5 (7.94)

Forty-five reception staff attended training (range 6–20). Analysis of appointments data revealed lower than expected increases in matching and use of telephone consultations (Table 3b, further information available from the authors on request). Analysis of field notes from observations of appointment-making (*n* = 4), and transcripts from GP (*n* = 19), and staff (*n* = 7) interviews representing all study practices found that matching was hampered by lack of flexibility in appointment-making systems, restrictions on GP availability, and patient preferences. Barriers to telephone consultations included GP preferences for face-to-face consultations and practice policies on usage, restricting slot length and number of slots available per day.

**Table 3b:** Proportion of matched and telephone consultations with eligible patients across groups

Using consultation data				
		**Before, %**	**After, %**	**Difference, %**
Proportion of consultations with named GP	Intervention	28.14	33.64	5.50
	Control	21.91	22.93	1.02
Proportion of telephone consultations	Intervention	29.87	33.37	3.50
	Control	44.34	48.93	4.59

Analysis of recorded BATHE consultations (*n* = 21) revealed adaptations in question composition and deviations from the training protocol.^25^ Tailoring of questions to individual patient circumstances were considered successful adaptations. However, adaptations changing the nature of the questions and deviations in question location were consequential for theoretical fidelity to BATHE, and thereby its potential effectiveness. Delivering BATHE ‘successfully’ gave patients the opportunity to share the wider context to their problems, and gave GPs opportunities to support patient self-management. Five recordings were made in control practices and there was no evidence of contamination.

Findings from the practice data audits and consultations analysis were fed back at top-up training sessions. Attending GP top-up trainings was associated with increased usage of BATHE. Despite GPs valuing BATHE, and many feeling they had discovered new information about their patients, they did not adopt the approach wholesale, preferring to selectively use it at times they considered appropriate. At interview, patients (*n* = 16) were positive about increasing continuity of care, but noticed little change in GPs’ consulting behaviour.

### Economic evaluation

Overall, complete cases of patient health and social care resource use data were collected from 55 (57.3%) participants. However, the proportion of missing data points was considerably less than implied, and in the analysis of a full trial, all available data would be used in an imputation model. The most common items reported were outpatient visits and the purchase of over-the-counter medication. Resource use data extracted at 12 months from EMRs were complete for 95/96 (99.0%) participants. Study patients were prescribed a mean of 15.2 medications each (range 1–42) and received 1775 tests, which were most commonly full blood counts (10.4%) and liver function tests (8.3%). The overall mean consultation length was 15.2 minutes and there was very little difference between control and intervention groups (14.8 versus 15.3 minutes), and between BATHE and non-BATHE consultations (14.6 versus 15.3 minutes).

The intervention cost was estimated at £5627 per practice. GP time accounted for over half of all cost; one-third of this time was for screening patients and two-thirds for training.

## Discussion

### Summary

It has been demonstrated that it is feasible and acceptable to run a trial of this primary care intervention. GPs identified >50% of their top 3% attenders as meeting the study inclusion criteria. The number of patients agreeing to be actively involved with the research was low. Patient retention rates were initially high but declined over time. Completeness of outcomes data collected was generally good. GP use of BATHE was lower than expected (although owing to lack of prior data no target was set a priori) and analysis of consultation recordings revealed variation in adherence to training. Analysis revealed some movement towards higher levels of patient activation, and lower consulting rates in the intervention group. The intervention was acceptable to staff, GPs, and participating patients at interview.

### Strengths and limitations

Practices were keen to take part in the study, and motivation among GPs to make service changes was generally high. The intervention was relatively low cost. Although it is not known how often GPs use elements of BATHE generally, the technique was found to be new to the majority of participating GPs. Although, during training, some reported fear of ‘opening a can of worms’ in a time-limited environment,^26^ use of BATHE did not seem to significantly increase consultation length. GPs reported BATHE to be a helpful tool to structure discussion about the wider context to patients’ problems and gave examples of it yielding new insights, even from patients they felt they knew well.

Data collection on consultation rates were limited to daytime GP contacts only. The feasibility of collecting data on use of out-of-hours primary care services, or the emergency services, were not assessed. Imbalances were noted between groups in age, sex, and deprivation level. This is likely to be due to the small number of clusters in this feasibility study. Owing to system-level issues, changes in continuity of care and use of telephone consultations were challenging to implement. Some GPs were unable to attend BATHE training sessions and uptake was lower than expected. However, the process evaluation has highlighted clear ways to optimise GP training.

### Comparison with existing literature

FAs were defined as patients falling within the top 3% of attenders during a 12-month period. Other studies have used a lower threshold (<25%) or an integer threshold (ranging from 6 to ≥12 contacts), and different periods during which contacts are counted (for example, 12 months or 24 months).^4^ All contacts with GPs were included, whereas other studies have included nurse and other staff contacts and excluded practice-initiated contacts. In contrast to a focus on ‘treatment’ of a sub-group of FAs (for example, patients with medically unexplained symptoms), the intervention aimed to support GPs to improve care for all. Although in this study patient recruitment was low, it exceeded that achieved in other studies with this population.^8^

There are other consultation models in use in GP teaching and/or practice that encourage a patient-focused approach; for example, the ideas, concerns, and expectations model.^27^ However, there is scant evidence for extent of use or efficacy.^28^ Uniquely, BATHE is an informal screening and therapeutic tool to support person-focused care. Two previous studies testing doctors' use of BATHE versus usual care with unselected patients demonstrated increased patient satisfaction post-consultation.^29,30^ More recently, a cluster randomised trial of BATHE versus usual care focusing on patients with diabetes mellitus showed a positive impact on empowerment to self-manage their condition.^31^ This is the first study to test the feasibility of training UK GPs to use BATHE.

### Implications for practice

It has been demonstrated that it is feasible and acceptable to run a trial of this primary care intervention. If a strategy were put in place to address key barriers to uptake, this intervention could be associated with increased support for patient self-management, lower consultation rates, and cost savings. The data collected have been used to create an optimised BATHE training package. Better training will improve GP confidence and competence, leading to higher fidelity in delivery. Online training resources are being developed by the authors to improve access to training and further reduce intervention costs.

One key uncertainty in this study was the choice of primary outcome measure. It is believed that a single outcome may be insufficient to describe all the potential effects of this complex intervention. A small effect on patient activation levels and consultation rate was observed and, going forward, retaining both is recommended. Further evidence of effectiveness is now needed in an adequately powered trial.

## Trial registration

This trial was registered with the ISRCTN registry (reference number: ISRCTN62939408).
